# Establishment of the emergency material reserve mechanism for public health emergencies and optimization of the management of various functional departments

**DOI:** 10.3389/fpubh.2022.1092375

**Published:** 2023-01-11

**Authors:** Yumeng Sun

**Affiliations:** School of Public Administration, South China University of Technology, Guangzhou, Guangdong, China

**Keywords:** public health events, emergency response, material reserve, optimization assembly, material reserve mechanism

## Abstract

Public health emergencies refer to emergencies caused by various factors that may cause serious harm to society. This paper mainly discusses how to establish an emergency reserve mechanism for public health emergencies. This paper proposes a comprehensive evaluation system for emergency response capabilities based on analytical methods, and analyzes the emergency response to public health emergencies and various functional management departments. The experimental results show that the implementation rate of disease prevention and control projects in the city center is 59.3%, and the average completion rate of inspection projects by district and county health supervision agencies is 45.8%. However, these achievements are far from enough. Therefore, the training of relevant personnel should be strengthened and run through the material reserve work from beginning to end. At the same time, an emergency material reserve system should be established. According to actual needs, timely provide emergency disposal materials to ensure normal supply. In response to the current public health emergencies, multiple departments and units should further strengthen management personnel allocation and optimize work processes to promote the development of public health.

## 1. Introduction

Public health emergencies refer to the hazards to public health caused by the outbreak of major infectious diseases, mass diseases of unknown causes, major food, occupational poisoning and other situations that endanger public health. Broadly speaking, it refers to the outbreak of major infectious diseases, infectious diseases of unknown causes, new infectious diseases, mass reactions of vaccines, mass drug reactions, major food poisoning, major environmental pollution, acute occupational poisoning, radiation pollution, radiation accidents, biological, chemical, nuclear radiation, major animal infectious diseases, natural disasters, accidents, public security and other emergencies. It has the characteristics of sudden, unexpected, mass, public, high-frequency, diversified, international, comprehensive, systematic, etc. It has the characteristics of multi disaster, disastrous and destructive. The sudden public health events not only endanger the public's physical and life safety, but also may cause psychological panic, even cause social chaos, economic recession, and threaten national security. The emergency response mechanism for public health emergencies of the CDC cannot be completed overnight, but is a long process. In the process of establishing the emergency system, it is necessary to conduct timely research and evaluation to understand the current situation and existing problems, and formulate corresponding countermeasures according to these problems to ensure the effectiveness of the system construction and operation, so as to effectively improve the work efficiency. This article discusses how to build the reserve mechanism of emergency disposal materials and the best management mode of each functional department in public health emergencies, with a view to making certain contributions to public health emergencies.

According to the existing research progress, different researchers have also conducted corresponding cooperative research on public health events. Li et al. (1) conducted an in-depth study on the spatio-temporal distribution characteristics and related influencing factors of Internet users' perception of public health event risk. Pan et al. (2) analyzed the main events and intervention methods of the COVID-19, and assessed the correlation between public health intervention measures and the epidemiological characteristics of COVID-19 epidemic in Wuhan in five periods. The concept of acute public health events usually assumed that they were resolved entirely or mainly through technical and medical solutions. In order to better understand this connection, Whittaker et al. (3) used the perspective of disaster diplomacy to analyze and evaluate the impact of acute public health events on diplomatic outcomes. McCullough et al. (4) estimated the direct cost of carrying out emergency preparedness and response activities simultaneously to deal with three major public health events. However, these scholars did not analyze the emergency response to public health events. In this regard, relevant documents on emergency handling events were consulted.

Some scholars also have some research on emergency response. Rose et al. (5) highlighted the development of public health emergency management in recent years, and discussed many cross issues in public health and emergency management. Public health events have caused great harm. Emergency management requires the strength of government departments, pharmaceutical enterprises, citizens, new media and other forces. Therefore, what are the effects of different choices of citizens and news media in responding to emergencies? In response to the above problems, Lu et al. established a four-dimensional evolutionary game model, which divided two types of citizen participation models into two categories: real assessment and false assessment. There are two ways for new media to participate: one is confirmed reports, the other is unconfirmed reports (6). However, these scholars did not discuss the establishment of the emergency response material reserve mechanism for public health emergencies and the optimization of the management of various functional departments, but only unilaterally discussed its significance.

This paper drew the following conclusions by studying and analyzing the optimization experiment results of public health emergency response and management of various functional departments. In order to deal with public health emergencies, effective measures should be taken to strengthen the management of relevant personnel. The regular material preparation system was established. According to actual needs, sufficient emergency disposal materials were arranged to ensure their normal supply.

The innovations of this paper are as follows: (1) The emergency handling material reserve process is explained, and the comprehensive evaluation method of emergency response capability based on the analytic hierarchy process is proposed. (2) The emergency response of public health events and the optimization of the management of various functional departments are experimentally analyzed.

## 2. Establishment of emergency response methods for public health emergencies

### 2.1. Emergency disposal material reserve process

The reserve of emergency handling materials for public emergencies refers to the reserve requirements for various emergency handling materials and equipment required to prevent and control public emergencies or disasters, including tents, folding beds, disinfection cabinets, sanitary toilets, emergency vehicles and other emergency handling materials. The preparation and implementation of the reserve plan for emergency materials shall include the types, quantities and storage methods of materials required. The types of emergency materials include: general protective articles, equipment and equipment, emergency equipment and materials, medical equipment, other living articles and other urgently needed items.

**Figure 1:**
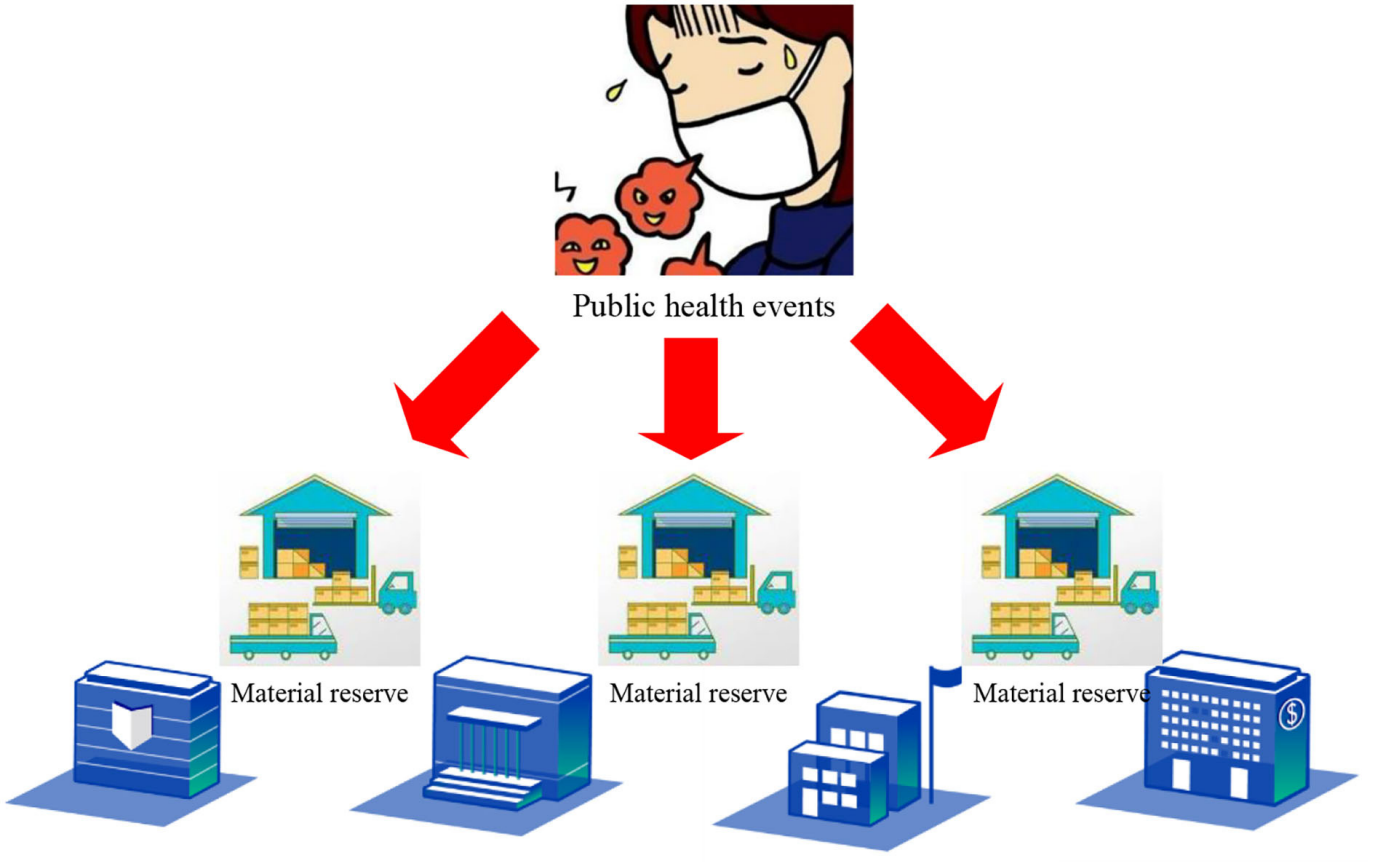
Full coverage of emergency materials reserve.

First of all, governments at all levels should establish an effective emergency materials reserve mechanism to effectively implement the emergency materials management system and achieve full coverage of emergency materials reserve for public health emergencies. At the same time, it has formed a complete and efficient management system (7, 8). Figure 1 shows the full coverage of emergency materials reserve. Secondly, governments at all levels should implement a centralized supply system of unified planning and allocation for emergency materials to form a unified centralized procurement system for emergency materials and implement a whole process tracking and supervision system. In addition, governments at all levels should also evaluate and supervise the reserve of emergency materials to form an evaluation and supervision management system and implement a whole process tracking and supervision system. The public purchase mechanism of emergency materials has been established to ensure that the market mechanism plays an effective role. The training mechanism for emergency materials management personnel has been established to ensure that they can allocate and use emergency materials in accordance with the law. The emergency materials management information system has been set up and its operation status has been disclosed to the public in a timely manner to facilitate the public's inquiry, supervision and management of the emergency materials reserve (9, 10). Finally, it is necessary to establish a communication and coordination mechanism among various government departments to timely communicate and solve the problems and difficulties in the work of emergency handling material reserves. Adequate supply of various materials is the precondition to ensure efficient and orderly disposal of public health emergencies. Emergency disposal material reserve is one of the important links to maintain social stability (11, 12). It is not only the material reserve itself that plays a decisive role in the disposal of public health emergencies, but also whether each department in the coordination mechanism of emergency material reserve can achieve information communication, personnel integration, material allocation, etc.

### 2.2. Strengthen organization and leadership to form resultant force

A sound emergency disposal material reserve system has been established. Relevant systems have been developed according to relevant requirements, and organizational leadership has been strengthened. A leading group consisting of the Municipal Health Commission, the Municipal Bureau of Commerce, the Municipal Big Data Bureau and other member units is established, and a regular meeting system is established to study and solve the problems in the emergency response reserve work. The organization and leadership system has been established and improved, and the public health emergencies, response processes and standards have been clarified. The responsibility is implemented, and the linkage mechanism of various departments is established to form the leadership system and working mechanism for responding to public health emergencies. The supervision and inspection of the medical protection material reserve is carried out regularly. The inspection results are included in the scope of responsibility assessment for the year-end objectives of the unit, forming a supervision and restraint mechanism, which can promote the relevant departments to effectively carry out the management of medical protection material reserves within their responsibilities. Work meetings are held regularly to study and deploy relevant work.

### 2.3. Comprehensive evaluation of emergency response capability based on analytic hierarchy process

The evaluation of emergency response capability is the basic evaluation of the emergency management system. It is a comprehensive evaluation of the possible disaster accidents, natural disasters and other natural disasters when emergencies occur. It also is a strategic decision that the country should make in response to emergencies in emergency management.

Calculation of index weight: Analytic Hierarchy Process (AHP) is adopted, and the influence degree of each index is divided into multiple levels to determine its interdependence. Considering the operability of the actual evaluation, a hierarchical index system is established. On this basis, the evaluation matrix is established by evaluating the relationship between the indicators, and the weight of each indicator is calculated according to the following formulas.


(1) $$\begin{array}{l} E_{o}^{{}^{\prime }}=\sqrt[{z}]{s_{o1}s_{o2}\cdots s_{oz}} \end{array}$$

(2) $$E_{o}=\frac{E_{o}^{{}^{\prime }}}{\sum_{o=1}^{z}E_{o}^{{}^{\prime }}}$$

In the above formulas, $E_{o}^{{}^{\prime }}$ is the initial weight coefficient and *E*_*o*_ is the normalized weight of the indicator.

Consistency test and overall consistency test are conducted for the acceptability of index weight coefficients. The consistency ratio (CR) is less than 0.1, and the consistency is relatively satisfactory.

The consistency index (CI) is calculated as follows:


(3) $$\begin{array}{l} CI=\frac{\bar{\gamma }-z}{z-1} \end{array}$$

(4) $$\bar{\gamma }=\frac{\sum_{o=1}^{z}\gamma_{o}}{z}$$

(5) $$\gamma_{o}=\frac{\sum_{k=1}^{z}s_{ok}E_{k}}{E_{o}}$$

In the above formulas, *z* is the number of sub targets of the inspected level. $\bar{\gamma }$ is the average of *z* characteristic roots. γ_*o*_ is the o-th characteristic root of the comparative judgment dominance matrix of the sub targets at this level. *s*_*ok*_ is the corresponding eigenvector.

The random consistency ratio CR is calculated as follows:


(6) $$\begin{array}{l} CR=\frac{CI}{RI} \end{array}$$

RI is a random index of the decision matrix.

In order to prevent the accumulation of minor inconsistencies from causing serious inconsistencies, it is also necessary to combine the consistency check with the overall consistency check. Let the consistency ratio of the first level judgment matrix be *CR*_(1)_ and the weight vector of the first level index be (*n*_1_, *n*_2_, ⋯*n*_*m*_). The consistency indexes of the second judgment matrix are *CI*_1_, *CI*_2_, ·*CI*_*m*_. The order of the second judgment matrix is *y*_1_, *y*_2_, ·*y*_*m*_. The consistency ratio of the portfolio is:


(7) $$CR_{\left(2\right)}=\frac{\sum_{o=1}^{m}n_{o}CI_{o}}{\sum_{o=1}^{m}n_{o}RI_{\left(y_{o}\right)}}$$

In the above formula, *RI*_(_*y*__*o*_)_ is the random consistency index of positive and negative matrices of order.

The formula for calculating the overall consistency ratio is:


(8) $$\begin{array}{l} CR^{{}^{*}}=CR_{\left(1\right)}+CR_{\left(2\right)} \end{array}$$

**Figure 2:**
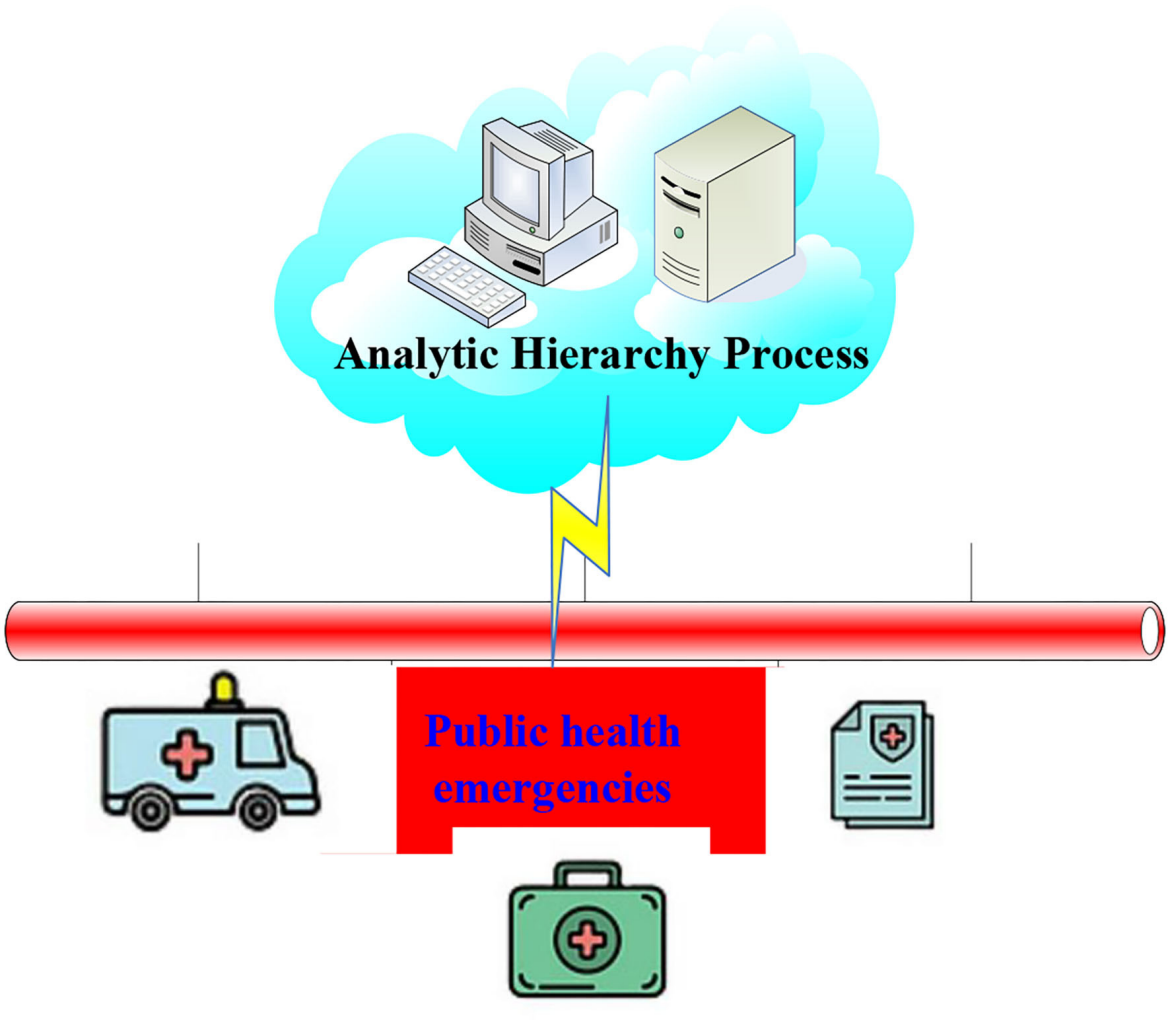
Application of analytic hierarchy process in public health emergencies.

The evaluation score of emergency response capability for public health emergencies is a comprehensive evaluation of the emergency response capability of medical institutions, and its comprehensive evaluation score reflects the response capability of medical institutions during this period (13, 14). Figure 2 shows the application of AHP in public health emergencies. When establishing the evaluation system of emergency response capability by establishing a calculation model to determine the participation of each subject, the hierarchical structure and hierarchical weight are analyzed and compared. The adoption of hierarchical structure and hierarchical weight calculation method makes each object have good consistency, which reflects the internal relevance and variability of each subject in the field of emergency management, thus making the evaluation results more comprehensive and objective.

On this basis, the weighted linear method is used to calculate the product of the weight of each index and the measurement results, and the comprehensive evaluation index is obtained. The formula is as follows:


(9) $$\begin{array}{l} W=\sum_{o=1}^{m}Q_{o}•R_{o} \end{array}$$

In the formula, *W* is the comprehensive evaluation index of the emergency response capacity of urban prevention and protection stations for public health emergencies. *Q*_*o*_ is to calculate the score of each index according to the dimensionless method. *R*_*o*_ is the comprehensive weight of the evaluation index. M is the number of evaluation indexes participating in the evaluation.

AHP method is used to establish the evaluation index system of emergency response capability. Through careful analysis of the indicators, and according to the actual situation, the subordination of each indicator is determined, and the correlation of each indicator element is noted to meet the attribute requirements of the upper level indicators. AHP method is used to study the evaluation index of emergency response capability, which fully reflects the idea of combining qualitative and quantitative methods in system theory.

## 3. Experimental results of optimization of public health emergency response and management of various functional departments

### 3.1. Emergency management mechanism

The establishment of material reserve mechanism for handling public health emergencies is the first step to ensure epidemic prevention and control. On the one hand, it is necessary to ensure national unity and centralized and unified command of authority. In the face of public health emergencies, the state should adopt a unified allocation model. On the other hand, it is necessary to establish a set of systems to plan, coordinate and manage the governments at all levels from the national level. The government and market entities shall specify the responsible subjects for providing material support after public health emergencies. At the same time, it should be made clear that the state should organize and carry out corresponding emergency response work immediately after the public health emergency. The establishment and management process of emergency response material reserve mechanism can improve the efficiency and effectiveness of all departments in dealing with public health emergencies (15, 16). The second step is that the government should make corresponding planning, design and arrangement according to the requirements of emergency response work to formulate relevant policies and regulations. It is ensured that the tasks of governments at all levels and departments are not absent or offside, which requires responsibility and accountability. The emergency response work should be effective and quality. The management should be open and transparent, free from blind spots and loopholes, and not affect the overall situation. This paper is illustrated by M City.

**Figure 3:**
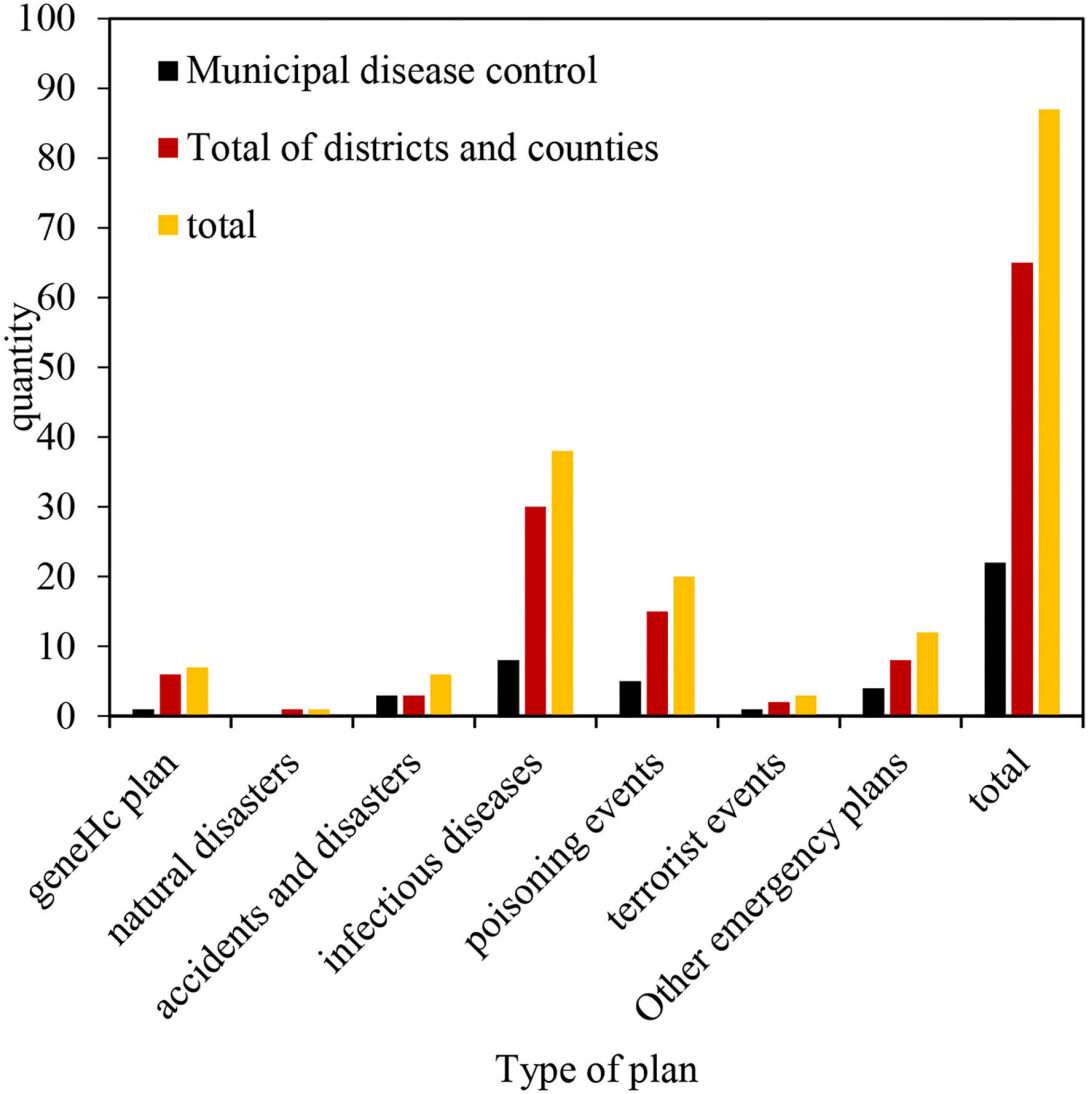
Plan formulation of disease prevention and control institutions in M City.

Disease prevention and control institutions in M City have corresponding emergency response and technical plans, including the overall plan and six special plans, totaling 87. Among the emergency plans formulated in the city, 38 are the emergency plans for public health emergencies caused by infectious diseases. Twenty are the emergency plans for poisoning events. Twelve are the emergency plans for other types of public health emergencies. There are seven overall plans, six emergencies, three terrorist events, and one natural disaster. As shown in Figure 3, it is the emergency plan of M City Center for Disease Control (CDC).

### 3.2. Basic information of health emergency team

(1) Establishment of health emergency team

**Figure 4:**
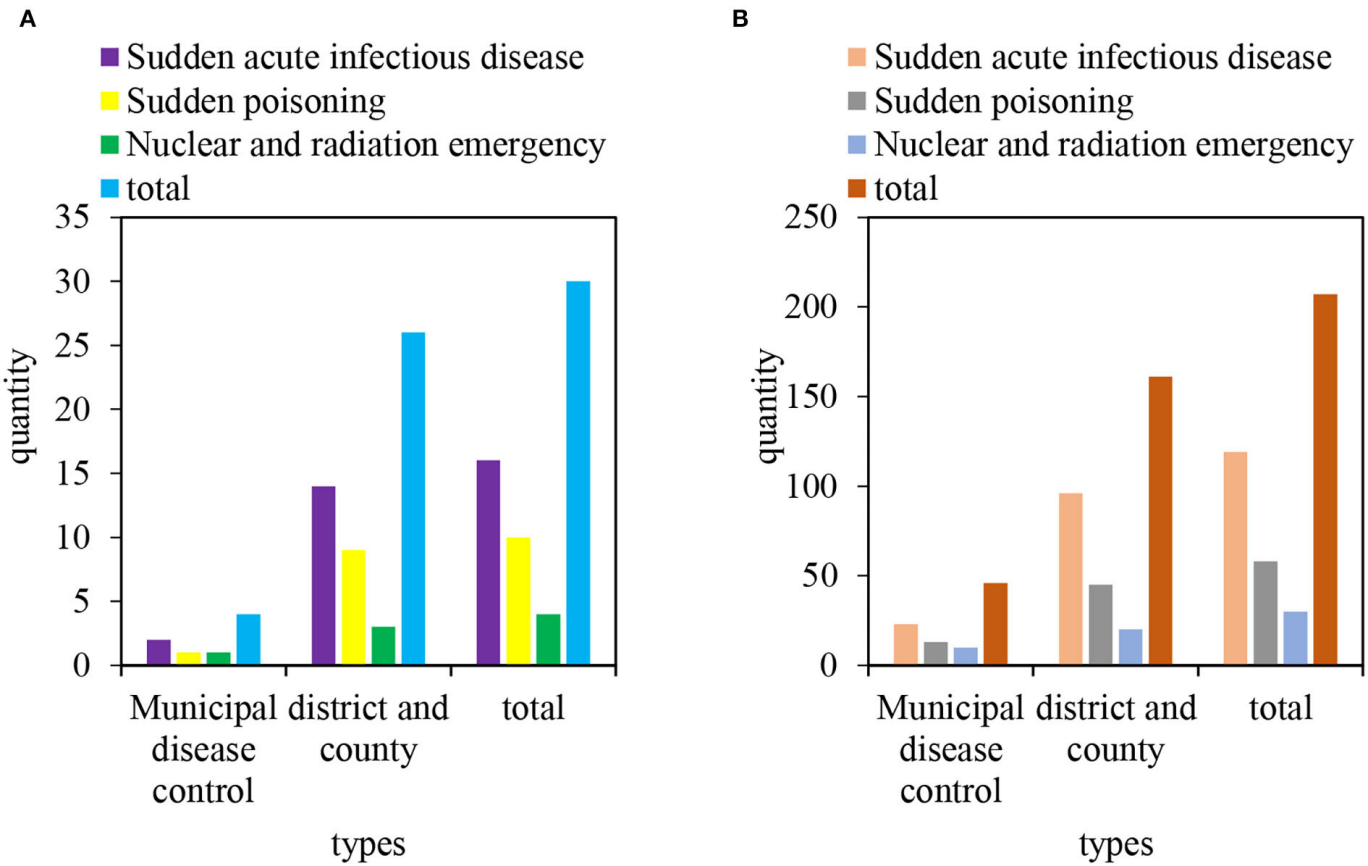
Establishment of health emergency response team of disease control institutions in M City. **(A)** Number of teams. **(B)** Number of personnel.

As of the end of 2018, there are 207 medical emergency teams in M City CDC. Among them, there are 16 emergency infectious disease prevention and control teams with 119 people. There are 10 disposal teams of 58 people for poisoning emergencies. There are 4 medical emergency teams with 30 people for nuclear radiation emergencies. Among them, there are 46 people from 4 departments of the municipal CDC and 161 people from district/county CDC. There are 14 emergency response teams for sudden acute infectious diseases, 9 emergency response teams for sudden poisoning, and 3 emergency response teams for nuclear and radiation. It can be seen that the construction of the emergency response team is dominated by sudden acute infectious diseases and sudden poisoning accidents, and there is a lack of attention to the emergency response work of nuclear and radiation accidents. As shown in Figure 4, Figure 4A is the number of teams and Figure 4B is the number of personnel.

(2) Age distribution

The health emergency workers of the CDC in M City are mainly concentrated in people aged 40~50, accounting for 52.17%. The population aged 30–40 years accounts for 30.44%. The proportion of people under 30 years old is 12.08%. 5.13% are over 50 years old. Among them, 14.91% are under 30 in district/county disease control units. The population aged 30–40 years accounts for 32.92%. The population aged 40–50 years accounts for 49.07%. The population aged over 50 accounts for 3.1%, as shown in Table 1.

**Table 1:** Age of health emergency personnel in disease control institutions in M City.

		**Municipal disease control**	**District and county disease control**	**Total**
Under 30	Number of people	1	24	25
	Proportion	2.17%	14.91%	12.08%
30–40	Number of people	10	53	63
	Proportion	21.74%	32.92%	30.44%
40–50	Number of people	29	79	108
	Proportion	63.05%	49.07%	52.17%
Over 50	Number of people	6	5	11
	Proportion	13.04%	3.1%	5.31%

### 3.3. Health emergency fund guarantee

The main source of emergency funds is local government funds. Emergency funds are mainly used for emergency disposal (investigation, disposal and inspection of emergencies), as well as storage of emergency materials (including antibacterial drugs, protective equipment, field equipment, communication equipment, etc.), and daily emergencies (training, drilling, and preparation of plans).

**Figure 5:**
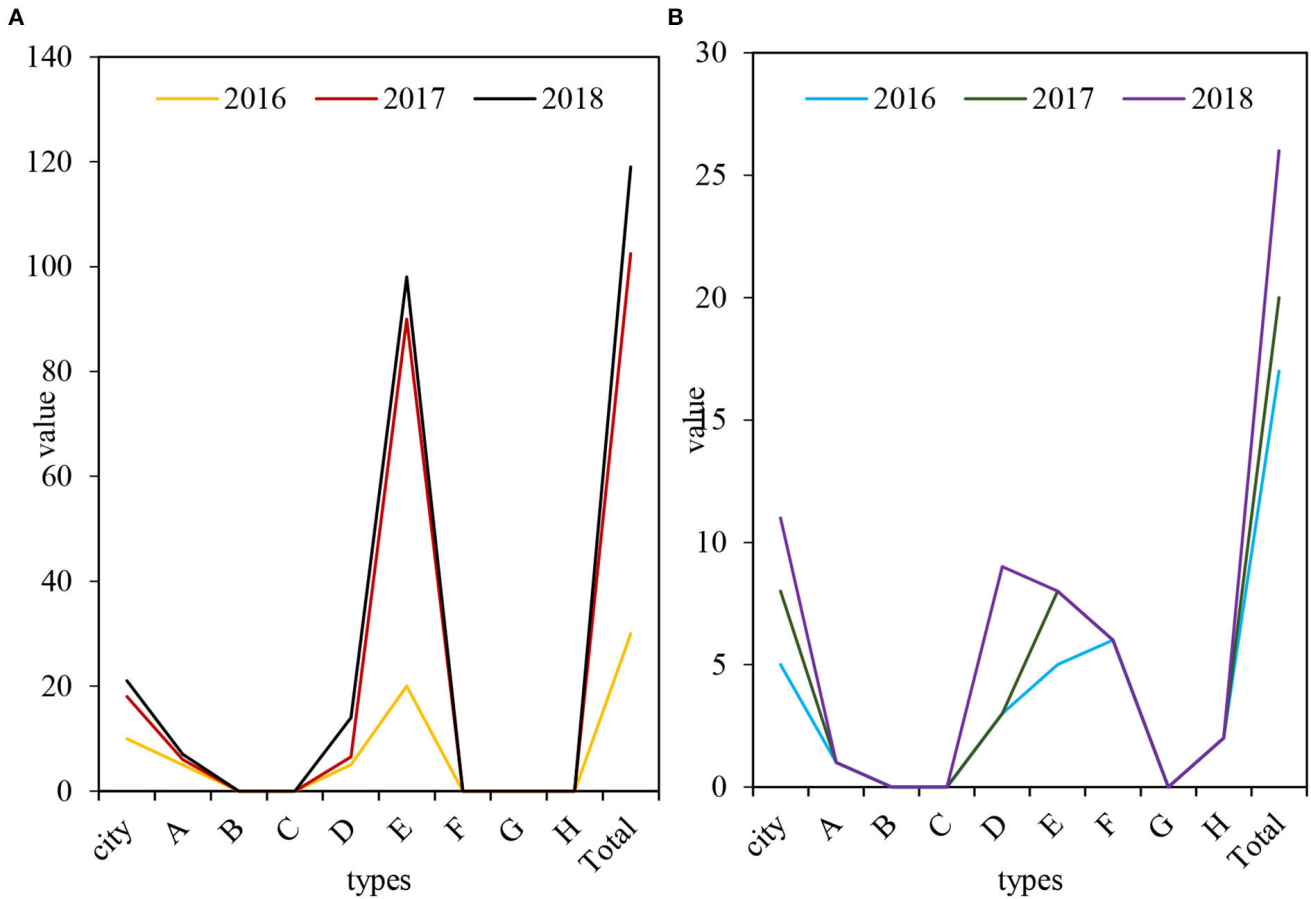
Emergency funds of disease control institutions in M City from 2008 to 2010. **(A)** Daily work fund, **(B)** Health emergency stockpile.

Among the eight centers for disease control and prevention, only the municipal centers for disease control and the four centers A, D, E, and H have included medical emergency funds and medical emergency reserve funds in their annual budgets every year. From 2016 to 2018, the daily funds of the CDC in the city are 1,00,000, 1,80,000, and 2,10,000 yuan, and the emergency reserves are 50,000, 80,000, and 1,10,000 yuan. The funds of district and county disease control departments are 3,00,000 yuan, 1.025 million yuan, 1.19 million yuan, and the emergency reserves are 1,70,000 yuan, 2,00,000 yuan, and 2,60,000 yuan, showing an increasing trend year by year. According to the survey results, the expenditure amount of each department is compared. Most of the Centers for Disease Control and Prevention have included the emergency fund in their financial budgets. They believe that the current emergency disposal costs simply cannot meet the needs of actual work. Only a few people feel that this is just a barely able to adapt to the requirements of work. As shown in Figure 5, Figure 5A is the daily work fund (10,000 yuan), and Figure 5B is the health emergency reserve (10,000 yuan).

### 3.4. Laboratory testing capability

**Figure 6:**
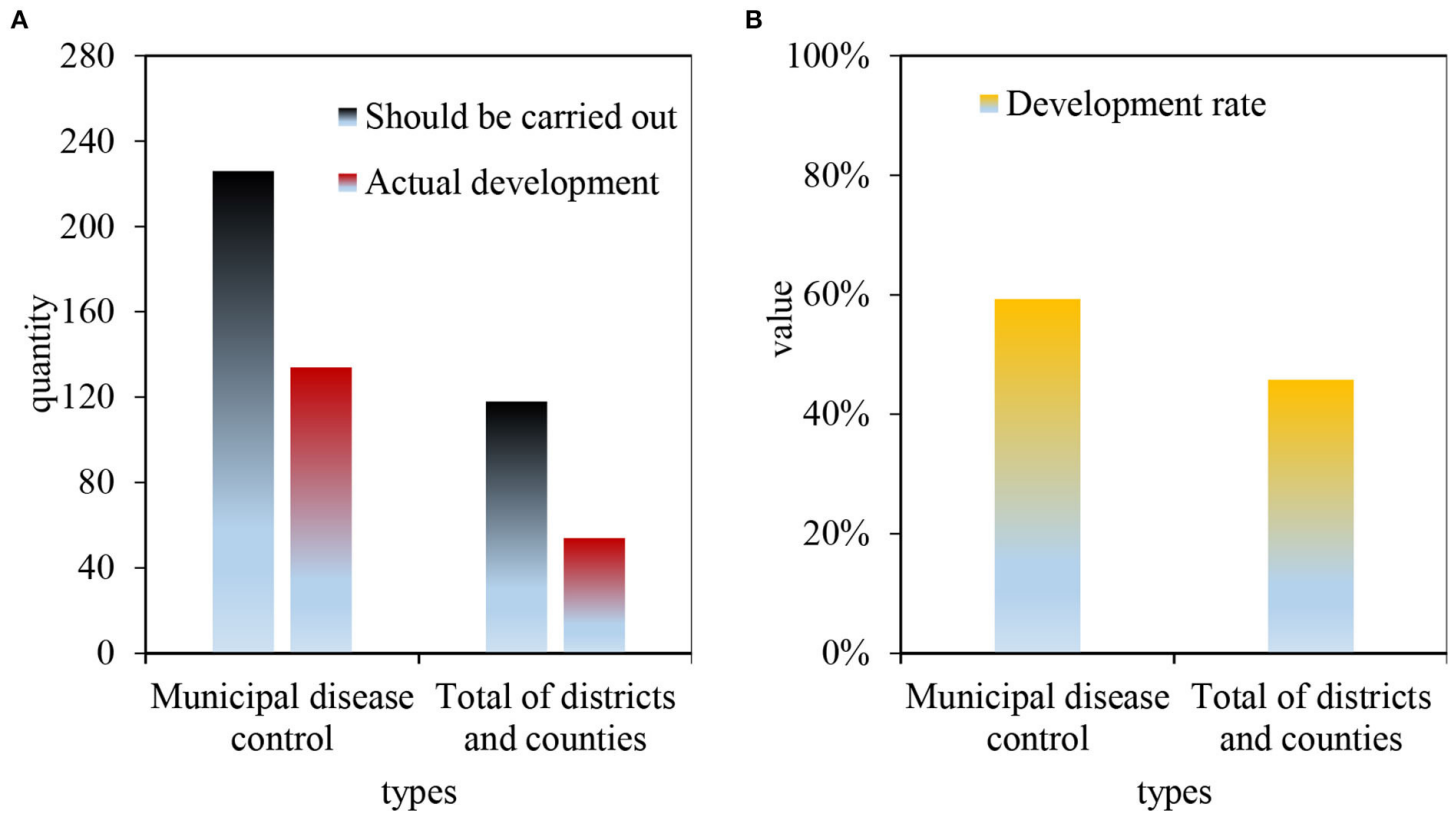
Work items that must be carried out by disease control institutions in M City. **(A)** Projects that should be carried out and actually carried out by the CDC in M City. **(B)** Project implementation rate of disease control agencies in M City.

It can be seen from Figure 6A shows the projects that should be carried out and actually carried out by the disease control institutions in M City. Figure 6B shows the project implementation rate of the disease control institutions in M City. There are 226 work projects that must be carried out by the municipal CDC, 134 of which are actually carried out, accounting for 59.3%. A total of 118 inspection items should be implemented by the district/county CDC, of which 54 items are actually implemented, with an average completion rate of 45.8%.

**Figure 7:**
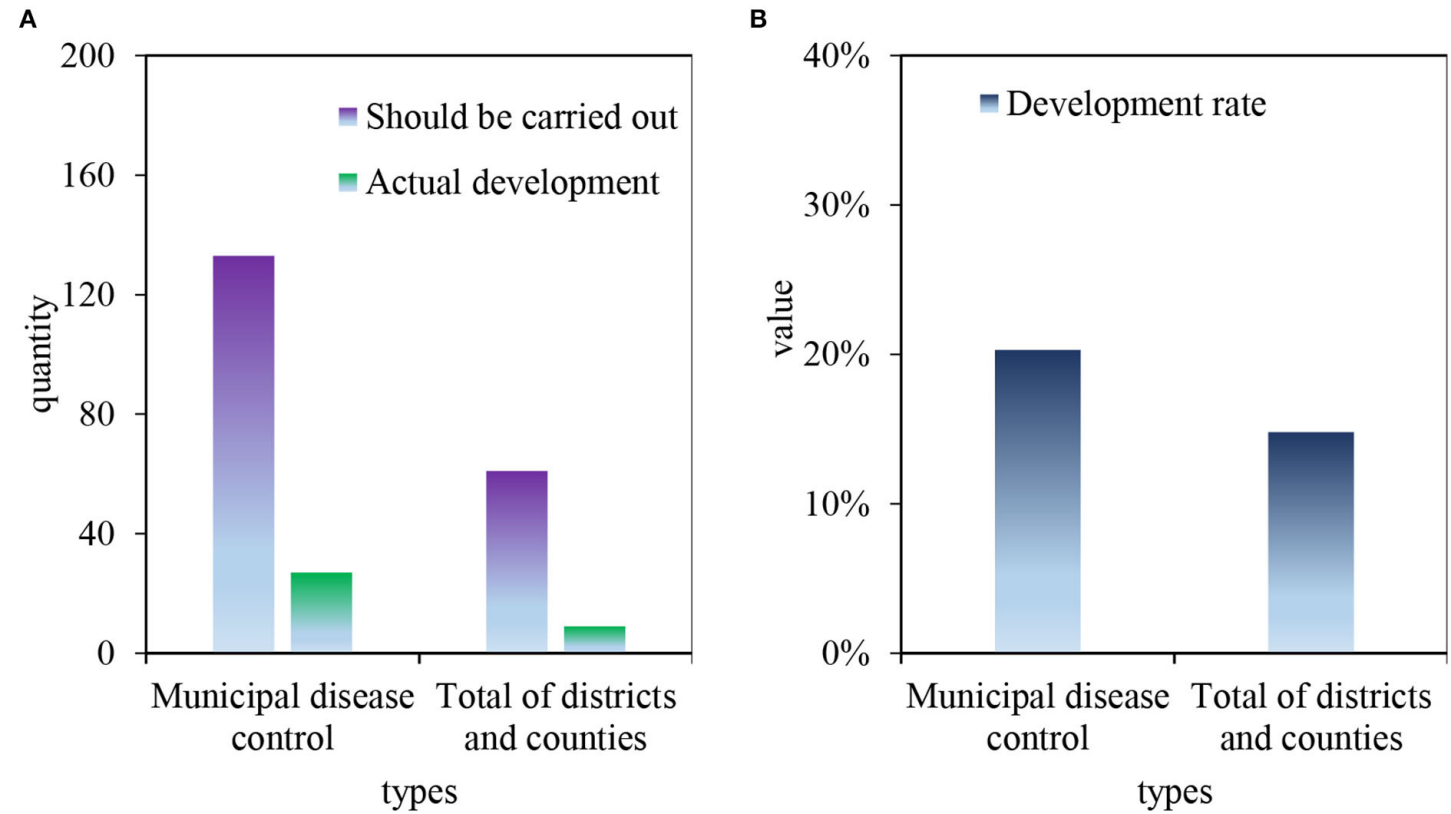
Work items to be carried out by disease control institutions in M City according to regional characteristics and needs. **(A)** The work items that the disease control agency of M City needs to carry out according to the regional characteristics. **(B)** The implementation rate of the work items that the disease control agency of M City needs to carry out according to the regional characteristics.

It can be seen from Figure 7A shows the projects that should be carried out and actually carried out by the disease control institutions in M City. Figure 7B shows the project implementation rate of the disease control institutions in M City. According to the regional characteristics and the work items to be carried out, 133 municipal disease control institutions should be completed. Among them, 27 projects are actually completed, accounting for 20.3%. District and county health management institutions should carry out 61 projects, and actually carry out 9 projects, with an average implementation rate of 14.8%.

### 3.5. M City's demand for emergency system construction of disease control institutions

It can be seen from Table 2 that the construction of the emergency system of the CDC in M City focuses on the construction of organizational structures (9), the increase of staff (9), the construction of troops and equipment (8), medical and emergency medical funds (9), emergency medical supplies reserves (7), the establishment of emergency measures (4), the treatment of emergency members (4), and the establishment of the emergency planning system (3).

**Table 2:** Recommended demand for health emergency system construction of disease control institutions in M City.

**Proposal**	**Number of institutions**
Strengthen the construction of health emergency organization and establish a full-time emergency office	9
Increase staffing and set up full-time health emergency management personnel	9
Unify equipment allocation standards and strengthen team and equipment construction	8
Set up special emergency funds for special purpose	9
Establish emergency reserve mechanism	7
Coordinate the government to establish operational emergency rules and regulations	4
Improve the treatment of health emergency team members and implement risk assurance	4
Improve plan construction	3

## 4. Discussion

Effective measures should also be taken to strengthen the management of relevant personnel in response to public health emergencies. First of all, a high-quality management team should be established. At present, many government departments and enterprises have different levels in the reserve management of emergency materials. In the process of formulating the reserve plan for emergency materials, it has not been implemented in strict accordance with the requirements of the provisions on the management of emergency materials. There are unscientific phenomena in the preparation of the reserve material plan. The emergency materials reserve plan blindly reserves materials without actual operation and use. Secondly, in the process of material storage, if the materials needed for some emergencies are not in place in time for handling, the emergency handling material storage plan cannot complete the actual consumption and disposal as required, affecting the normal production order. Therefore, the training of relevant personnel should be strengthened and run through the whole material reserve work.

A normalized material reserve mechanism is established to provide sufficient emergency materials according to the actual situation and ensure their normal supply. At the same time, corresponding work processes should be developed to sort out and optimize each link to ensure the orderly and effective operation of the whole process, so as to avoid excessive dispersion and waste. All departments should coordinate and cooperate with each other, and strengthen publicity and education, which gives full play to the role of public participation. The establishment of a scientific and effective mechanism to deal with public health emergencies is one of the important guarantees for doing all work well. For the problems and weak links exposed in the daily work of many departments and units in public health emergencies, it is also necessary to strengthen the allocation of managers and the optimization of work processes in these departments, so as to better promote the development of public health.

## 5. Conclusions

A long-term mechanism for emergency materials management should be established. At ordinary times, relevant materials shall be stored according to the needs and relevant standards. The implementation of material reserve has been checked regularly to find problems and report them in time. It is also necessary to do a good job in the delivery and management of emergency materials. Emergency materials shall be allocated and used by all functional departments. In the process of responding to public health emergencies, it is not only necessary to ensure the adequate supply of materials, but also to timely and accurately convey information to everyone, which ensures the timely distribution of materials in place to maximize their role. This must be fully considered, accurately designed and reasonably allocated to ensure the efficiency, safety and fair use of resources. After the establishment of emergency disposal donation warehouse, the management method for emergency disposal donation materials should be established and improved as soon as possible to clarify the division of responsibilities and operating procedures of all parties, and ensure the quality, safety and effectiveness of emergency disposal materials. At the same time, system documents such as operating procedures for the use of donated materials for emergency disposal and acceptance management measures should be developed and improved. The use process and relevant regulations of donated funds and materials shall be clarified, and the use specifications, management requirements and principles of donated funds and materials shall be clarified. At the same time, the management of donated money and materials has been strengthened to effectively improve the use efficiency of donated money and materials. However, due to the limitations of time and technology, this article did not elaborate on the problems encountered in the emergency response to public health emergencies, which would be further analyzed later.

## Data availability statement

The original contributions presented in the study are included in the article/supplementary material, further inquiries can be directed to the corresponding author.

## Author contributions

YS: writing—original draft preparation, editing data curation, and supervision.
